# SIRT1 in the Development and Treatment of Hepatocellular Carcinoma

**DOI:** 10.3389/fnut.2019.00148

**Published:** 2019-09-25

**Authors:** Marius Farcas, Andrei-Alexandru Gavrea, Diana Gulei, Calin Ionescu, Alexandru Irimie, Cristina S. Catana, Ioana Berindan-Neagoe

**Affiliations:** ^1^“Iuliu Hatieganu” University of Medicine and Pharmacy, Cluj-Napoca, Romania; ^2^Research Center for Functional Genomics, Biomedicine and Translational Medicine, “Iuliu Hatieganu” University of Medicine and Pharmacy, Cluj-Napoca, Romania; ^3^MEDFUTURE–Research Center for Advanced Medicine, “Iuliu-Hatieganu” University of Medicine and Pharmacy, Cluj-Napoca, Romania; ^4^5th Surgical Department, Municipal Hospital, Cluj-Napoca, Romania; ^5^11th Department of Oncological Surgery and Gynecological Oncology, University of Medicine and Pharmacy “Iuliu Hatieganu”, Cluj-Napoca, Romania; ^6^Department of Surgery, The Oncology Institute “Prof. Dr. Ion Chiricuţǎ”, Cluj-Napoca, Romania; ^7^Department of Medical Biochemistry, “Iuliu Hatieganu” University of Medicine and Pharmacy, Cluj-Napoca, Romania; ^8^Department of Functional Genomics and Experimental Pathology, The Oncology Institute “Prof Dr. Ion Chiricuţǎ”, Cluj-Napoca, Romania

**Keywords:** miRNA, cancer, HCC, sirtuin 1, cancer stem cells

## Abstract

Hepatocellular carcinoma (HCC) is one of the most common causes of cancer-related death worldwide. Current treatment options for inoperable HCCs have decreased therapeutic efficacy and are associated with systemic toxicity and chemoresistance. Sirtuin 1 (SIRT1) is a nicotinamide adenine dinucleotide–dependent enzyme that is frequently overexpressed in HCC, where it promotes tumorigenicity, metastasis, and chemoresistance. SIRT1 also maintains the tumorigenic and self-renewal proprieties of liver cancer stem cells. Multiple tumor-suppressive microRNAs (miRNAs) are downregulated in HCC and, as a consequence, permit SIRT1-induced tumorigenicity. However, either directly targeting SIRT1, combining conventional chemotherapy with SIRT1 inhibitors, or upregulating tumor-suppressive miRNAs may improve therapeutic efficacy and patient outcomes. Here, we present the interaction between SIRT1, miRNAs, and liver cancer stem cells and discuss the consequences of their interplay for the development and treatment of HCC.

## Introduction

Hepatocellular carcinoma (HCC) is the most frequent primary liver malignancy and among the most common causes of cancer-related death worldwide (1, 2). The majority of cases occur due to HCV or alcoholic cirrhosis (3). However, HCC can also develop in obese individuals with non-alcoholic steatohepatitis (NASH) (4–7). The increasing incidence in diet-induced NASH is estimated to upsurge the number of patients with NASH-related HCC (3). The main therapeutic options for HCC include liver transplantation, surgical resection, and chemotherapy (8). However, most patients present with advanced-stage, unresectable HCC. Moreover, first-line treatment compounds such as sorafenib have low response rates (9) and are associated with systemic toxicity and chemoresistance (10, 11). Therefore, a better understanding of the underlying mechanisms that promote HCC development, chemoresistance, and metastases is vital for improving patient outcomes (12).

Mammalian sirtuins (SIRT1-7) are NAD^+^-dependent deacetylases that are involved in a wide variety of biological processes including energy metabolism and lifespan and health span regulation (13). Mammalian sirtuins possess histone deacetylase, mono-ADP-ribosyltransferase, desuccinylase, demalonylase, demyristoylase, and depalmitoylase activity (14). SIRT1 is chiefly localized in the nucleus and plays a role in genomic stability, telomere maintenance, and cell survival (15, 16). SIRT1 regulates both histones and multiple downstream non-histone targets such as estrogen receptor-alpha (17), PPARγ (18), PGC-1α (19), androgen receptor (20), FOXO transcription factors (21), p53 (22), NF-κB (23), and Survivin (24). SIRT1 can also upregulate oncogenes: β-catenin (25), c-Myc (26), and HIF-1α (27) increasing their activity as a result.

SIRT1 is vital for the physiological function of healthy tissues. For instance, SIRT1 null mice have defects in hepatocyte metabolism and a shortened life span (28). SIRT1 deletion in mice hepatocytes results in hepatitis and hepatic steatosis (29). Oppositely, inducing SIRT1 activity in healthy tissues with synthetic activators or transgenic expression provided a plethora of benefits. SIRT1 overexpression reduced the release of pro-inflammatory cytokines and increased cell viability (23, 30, 31). SIRT1 also preserved the functions of hepatocytes and adipocytes against obesity (32). Overall, SIRT1 can be called a “Master Metabolic Regulator” (33), which is essential for normal hepatic function.

## The Expression and Function of SIRT1 in HCC

SIRT1 has a multifaceted relationship with oncogenesis. SIRT1 is overexpressed in multiple malignancies, including human myeloid leukemia (34), colon cancer (35), prostate cancer (36), and squamous cell carcinomas (37). Conversely, SIRT1 expression is reduced in ovarian cancers and glioblastoma (38) when compared to corresponding normal tissues. Overall, SIRT1 may function as both an oncogene and tumor suppressor depending on subcellular localization, age, type of tissue, and concomitant mutations in related signaling pathways.

In HCC, SIRT1 was the only member of the Sirtuin family consistently overexpressed (39) and deemed vital for all stages of HCC tumorigenesis (39). Moreover, it was repeatedly demonstrated that SIRT1 was frequently overexpressed in HCC biopsies when compared to corresponding adjacent non-cancerous liver parenchyma (40–42) and its expression was necessary for the maintenance of HCC tumorigenesis (15, 43–45). Generally, SIRT1 mRNA levels are similar in HCC and non-malignant adjacent tissue, suggesting that SIRT1 is increased in HCC *via* a post-transcriptional mechanism (15). Hypermethylated in cancer 1 (HIC1) and p53 negatively regulate SIRT1 mRNA transcription and are often mutated or dysfunctional in HCC. Thus, SIRT1 overexpression may be partly accounted for by the decreased inhibition of its transcription. However, SIRT1 protein levels are also preserved post-translationally *via* reduced degradation and increased stability (15, 46).

Additionally, SIRT1 was overexpressed in a multitude of human HCC cell lines such as HKC1-4, SNU-423, HKC1-2, PLC5 SNU-449, SK-Hep-1, Huh-7, HepG2, and Hep3B (15, 45), when compared to normal liver cell lines (47).

However, there is still some controversy regarding SIRT1's role in HCC, as some reports showed that SIRT1 was downregulated in human HCC samples and hypothesized it had tumor-suppressive roles (38). The multifaceted role of SIRT1 in carcinogenesis suggests (48) that its function is dependent on cancer type and the state of downstream or upstream molecules that influence its oncogenicity (49). The role of SIRT1 in HCC may also depend on its subcellular localization. Although, in HCC cells, SIRT1 had a predominant nuclear localization where its expression promotes tumorigenesis, it was reported that cytoplasmatic SIRT1 may have tumor-suppressive roles (50).

Multiple lines of evidence suggest that SIRT1 expression has survival-promoting effects in both normal hepatocytes and in HCC cells. In healthy mice, SIRT1 overexpression protected against malignancies (51) and basal SIRT1 expression was vital for maintaining physiologic hepatic morphology and normal lifespan (44). However, basal SIRT1 levels were lower in mouse livers compared to other viscera, indicating that the hepatocytes may be more sensitive to the under- or overexpression of SIRT1 (44).

Similarly, SIRT1 expression is vital for the proliferation and survival of HCC cells (44). Malignant cells were shown to enhance their function by hijacking survival signaling pathways of non-malignant cells (52, 53). Therefore, SIRT1 activity may promote cellular function and survival and inhibit cancerous transformation in normal hepatocytes; after malignant transformation, SIRT1's functionality may be employed in promoting tumorigenesis and sustaining HCC survival (15). That is, SIRT1's activity may promote cellular survival independent of the cancerous or non-cancerous state of the hepatocytes. As of yet, there are no reports of experimentally induced oncogenesis *via* SIRT1 overexpression. Finally, SIRT1 overexpression does not appear to be a cancer-initiating event but rather a cancer-induced adaptive mechanism that promotes survival and proliferation (42). However, because SIRT1 simultaneously regulates a wide spectrum of biological processes, its role in HCC oncogenesis is incompletely understood and further research is warranted in order to clarify at which level and *via* what mechanisms do HCC cells increase and become dependent on SIRT1 expression. Additionally, the interplay between SIRT1 and the other six sirtuin family members and their role in HCC should be further explored.

Multiple studies evaluated the prognostic value of SIRT1 expression in HCC. SIRT1 overexpression correlated with the development of portal vein tumoral thrombosis, decreased overall survival rates, lower disease-free survival, and advanced TNM stages (54). Patients with SIRT1-positive HCC biopsies had a decreased 10-year survival compared to SIRT1-negative HCC patients. SIRT1 protein levels appear to be positively correlated with HCC grades; specifically, SIRT1 expression is higher in advanced HCC stages. One meta-analysis investigated the prognostic and clinical implications of SIRT1 expression in HCC. It showed that heightened SIRT1 expression was associated with decreased patient overall survival and death-free survival. Moreover, increased SIRT1 expression correlated with larger tumor size, higher p53 expression, high alpha-fetoprotein (AFP) levels and advanced TNM stages (55). However, it was highlighted that, for the studies examined in the meta-analysis, there was no clear cutoff value or unified standard for the measurement of SIRT1 expression. Even though the statistical power was limited, it can be concluded that increased SIRT1 expression correlated with a poor HCC prognosis (26).

The deacetylation function of SIRT1 is vital for its oncogenic role in HCC. When the deacetylation domain of SIRT1 is mutated, the proliferation and colony formation ability of HCC cells are inhibited (40). Inhibition of SIRT1 in HCC cells, either through knockdown or administration of SIRT1 inhibitors, led to decreased tumor development *in vitro* and *in vivo* and exerted cytostatic as opposed to a cytotoxic effect (42, 44), while SIRT1 overexpression accelerated HCC growth (44). However, *in vivo* experiments indicate that other mutations in relevant cancer-related pathways might determine the function of SIRT1, thus, the role of SIRT1 should be viewed as context dependent (56). SIRT1 is also implicated in the malfunction of multiple HCC signaling pathways such as FOXO1, p53, and TGF (57–59). SIRT1 downstream targets involved in HCC progression include YAP (Yes-associated protein) (44, 60), PTEN/PI3K/Akt (61, 62), telomerase, and p53 (63). Overall, in HCC, SIRT1 acts as a potential oncogene (45). Further on, we will elaborate on the interplay between SIRT1 and the aforementioned pathways and molecules.

SIRT1 expression was also shown to prevent malignant development in a mouse model of metabolic-syndrome associated HCC. Communicable infectious diseases have been successfully dealt with in the past decades. However, in the early twenty-first century, non-communicable diseases have become a principal health hazard. The global spread of high calorie and low fiber, Western style foods, coupled with decreases in physical exercise led to a global epidemic of metabolic syndrome. The financial burden inflicted by metabolic syndrome is in the trillions (64). The epidemiology of obesity-associated HCC (65) and *in vitro* and *in vivo* experiments suggest that an obesogenic lifestyle, *via* pro-inflammatory cytokines, insulin resistance, steatosis, and lipotoxicity, may progress from metabolic syndrome to NASH (6) and HCC (65). Overall, diet-induced NASH is estimated to upsurge the number of patients with NASH-related HCC (3, 66–68).

SIRT1 expression promotes genomic stability in normal hepatocytes and appears to be protective against high-fat diet (HFD)-induced HCC. Moreover, the role of SIRT1 as a protector against metabolic syndrome is clear (69, 70). For instance, enhancing SIRT1 activity in a mouse model of type 2 diabetes leads to improved insulin resistance and controls hyperglycemia (7, 71, 72). Moreover, transgenic mice that systemically overexpress SIRT1 were protected from the hazards produced by a HFD (69, 73).

One model of metabolic syndrome-associated cancer examined the effects of a threefold systemic SIRT1 expression on diet-associated HCC.

Mice overexpressing SIRT1 systemically at approximately threefold that of the normal WT mice had measurably increased hepatic SIRT1 deacetylase activity. These mice had improved glucose tolerance, decreased adipose inflammation, and were protected from other negative effects of HFD such as hepatic steatosis. Moreover, compared to the control group, SIRT1-overexpressing mice displayed a lower incidence of HCC after the chronic administration of a HFD. Part of the protective effects of SIRT1 expression in HCC development was attributed to decreasing NF-κB-induced inflammation and malignant transformation (51).

Overall, systemic threefold SIRT1 overexpression protects hepatocytes but not fibroblasts from DNA damage and translates as safeguard against HFD-induced HCC (51).

SIRT 1 may promote protective effects against HCC *via* its effect on β-catenin (25) —an oncogene associated with epithelial cancer (74). This may account for the carcinoma-selective protection provided by SIRT1 overexpression.

Collectively, the current body of literature suggests that SIRT1 expression has a pro-tumorigenic role in HCC but is not a cancer-initiating event.

## Role of SIRT1 in the Tumorigenicity of Liver Cancer Stem Cells

Multiple models have been proposed in order to explain the functional and histological heterogeneity of solid cancers. One of them proposes a hierarchical organization of tumoral cell populations where a minor cell population termed cancer stem cells (CSCs) with self-renewal and differentiation capacities repopulate tumors and establish the histological and functional heterogeneity characteristic of most cancers (75, 76). Intra-tumoral CSCs are capable of differentiation and self-renewal and give rise to tumors identical to the original one in primary and metastatic sites (76, 77). HCC tissue samples possess intra-tumoral heterogeneity (78, 79), and a subpopulation of cells with stem cell-like proprieties might give rise to HCC and accelerate cancerous proliferation (80–82). Therefore, due to their proliferation and differentiation abilities, liver CSC (LCSC) have been incriminated for HCC initiation (83), chemoresistance (84, 85), metastasis (83), recurrence, and overall dismal patient outcome (83, 86–89).

For instance, hepatoblasts are cell progenitors with the ability to differentiate into hepatocytes (90). During chronic liver inflammation, hepatoblasts and other hepatic progenitor cells accrue genetic and epigenetic modifications, leading to their conversion in LCSCs (91). Importantly, through a process called dedifferentiation, hepatocytes can also undergo malignant transformation by acquiring CSC phenotypes (92). HCC CSCs can be identified through multiple biological markers such as CD133, CD90, and CD13 and ubiquitin-specific protease 22 (USP22) (82, 93). It was shown that SIRT1 plays a vital role in the self-renewal and maintenance of embryonic stem cells (94) and hematopoietic stem cells (95) and is also implicated in the biology of LCSC.

### SIRT1–MRPS5 Signaling Pathway

Liver cancer stem cells (LCSCs) use enhanced mitochondrial respiration to generate ATP. In contrast, cancer cells primarily rely on aerobic glycolysis (Warburg effect) to generate ATP. Metabolic reprogramming is a hallmark of cancer and plays a vital role in cancer progression (96); however, the regulator of metabolic reprogramming that drives the switch from oxidative phosphorylation to aerobic glycolysis in LCSC is not fully understood.

Mitochondrial ribosomal protein S5 (MRPS5) is required for the enhanced mitochondrial function of LCSCs and promotes cancer commencement and development (97, 98). Acetylation promotes the nuclear translocation of various proteins (99). SIRT1 is highly expressed in LCSCs (100, 100) where it deacetylates MRPS5, thus determining its subcellular localization. In LCSCs, deacetylated MRPS5 is located in the mitochondria where it promoted oxidative phosphorylation, stimulated NAD+ production, and improved mitochondrial function, thus preserving LCSC stemness. Increased NAD+ also maintains SIRT1 activity and promotes a SIRT1–MRPS5 positive feedback loop. In contrast, in HCC cells, acetylated MRPS5 translocates to the nucleus and consequently promotes metabolic flexibility and enhanced glycolysis (101).

Relevantly, multiple acetylated proteins act as metabolic enzymes in the extra nuclear environment and as transcription factors when located inside the nucleus (102). Nuclear translocation of MRPS5 led to enhanced expression of glycolytic proteins and a switch in metabolism, from oxidative phosphorylation to a Warburg-type metabolism. Thus, MRPS5 may function as a transcription factor when localized in the nucleus and consequently regulate the expression of glycolytic genes. However, the exact mechanism by which MRPS5 increases the expression of glycolytic proteins is not clear. Further research should establish whether acetylated MRPS5 acts as a glycolysis promoting transcription factor in HCC.

SIRT1 expression was higher in LCSC compared to HCC cells. The comparatively lower SIRT1 expression in HCC cells may account for the predominant nuclear localization of MRPS5. However, the tumor microenvironment is highly dynamic and SIRT1 expression may be heterogeneous in different cellular subpopulations at different time points. Thus, fluctuating SIRT1 expression may contribute to tumoral heterogeneity and self-renewal by facilitating the transition of LCSCs to HCC cells. This may explain why MRPS5 is found in higher concentration in HCC cell's nucleus even though SIRT1 is frequently overexpressed in HCC cells and it would be expected to deacetylate MRPS5 and thus promote its mitochondrial localization.

SIRT1 also induced the mitochondrial unfolded protein response (UPRmt) that preserves cell longevity and metabolic fitness (103, 104). LCSCs present accelerated oxidative phosphorylation, which is associated with increased ROS production. Therefore, the SIRT1–UPRmt axis maintained LCSC viability by reducing ROS.

**Figure 1:**
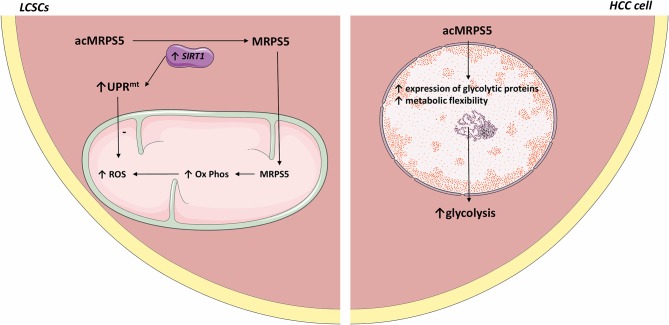
Comparative representation of MRPS5 signaling in liver cancer stem cells and HCC cells. In LCSCs, SIRT1 deacetylates MRPS5, which promotes its mitochondrial localization. Mitochondrial MRPS5 increases oxidative phosphorylation and consequently ROS. However, SIRT1 also promotes UPRmt activity, which decreases ROS levels. Conversely, in HCC cells, MRPS5 is localized in the nucleus where its activity promotes increased cellular glycolysis. All the figures in this article were made using images from http://smart.servier.com.

Metformin was reported to be beneficial to HCC patients (58). Interestingly, metformin downregulates MRPS5, which inhibits the activity of mitochondrial complex 1, thus decreasing the function of LCSCs (see Figure 1).

Human HCC samples with increased SIRT1 expression and high cytoplasmic MRPS5 levels presented more CSCs and were associated with high metastases rates, cancerous embolization, increased tumor size, and decreased survival compared to patients whose HCC biopsies showcased low cellular SIRT1 expression and high nuclear MRPS5 levels. This further indicates that the metabolic reprogramming induced by SIRT1/MRPS5 axis is crucial for the stemness of CSCs.

In summary, the SIRT1/MRPS5 axis augmented the metabolic plasticity and reprogramming of LCSC by ameliorating and maintaining mitochondrial function, consequently promoting hepatocarcinogenesis. Further studies should explore the interaction between SIRT1 and other MRPs. Administration of LCSC-targeted oxidative phosphorylation inhibitors or MRPS5 inhibitors should be explored in future experiments.

### SIRT1–SOX Signaling Pathway

*In vivo* mice models demonstrated that SIRT1 is necessary for maintaining the self-renewal and tumorigenicity of LCSCs. In those models, silencing SIRT1 expression reduced the incidence of HCC compared with the controls (100). SIRT1 is overexpressed in LCSCs where it is necessary for maintaining oncogenesis and self-renewal and is correlated with a poor prognosis of HCC patients.

The core embryonic transcription factor circuitry (SOX2, c-Myc, Oct4, Nanog) is implicated in the self-renewal of CSCs (105–107). SIRT1 induces tumorigenicity in a subpopulation of LCSC in a SOX2-dependent manner. SIRT1 knockdown in LCSCs decreased SOX2, Oct4, and Nanog expression levels. Treating LCSCs with SIRT1 inhibitors TV6 and EX-527 reduced SOX2 and Nanog (100).

SIRT1 regulates SOX2 gene expression thus primes LCSC for self-renewal. DNA (cytosine-5)-methyltransferase 3A (DNMT3A) catalyzes the transfer of methyl groups to CpG DNA structures. In LCSCs, SIRT1 expression inhibited DNMT3A, consequently promoting hypomethylation of the SOX2 promoter and activated SOX2 gene expression, consequently inducing self-renewal and oncogenicity (100). Notably, HCC stage and recurrence were correlated with SIRT1 and SOX2. Thus, in LCSCs, SOX2 is a prime downstream regulator of SIRT1-induced self-renewal and oncogenesis (100).

The Ubiquitin Proteasome Pathway (UPP) is the prime mechanism for protein catabolism in mammals. UPP is also responsible for degrading SIRT1 (108, 109). IGF1 was vital for the self-renewal and tumoral growth of LCSCs. Namely, IGF signaling inhibited the UPP pathway, thus increasing SIRT1 protein levels and function in LCSCs (100). Thus, IGF1 may enhance the self-renewal of LCSCs by mediating SIRT1 levels (100).

Inhibiting SIRT1 in LCSCs reduced SOX2 expression and strongly repressed tumor growth in both *in vivo* and *in vitro* models. Overall, SIRT1 deacetylase activity was vital for the oncogenicity and self-renewal of LCSCs. Selective SIRT1 inhibition in LCSCs is a potential therapeutic target hindering HCC development and progression.

### SIRT1–MEK Signaling Pathway

Initially, HCC heterogeneity was attributed to hepatocytes since the liver was presumed to lack a distinct stem cell population (110). Nevertheless, accumulating evidence shows that HCCs display multiple cell subpopulations, some of which have stem cell characteristics (111, 112), and increased expression of CSC1 markers (Nanog, SOX2, Oct4) was identified in some HCC subpopulations (113, 114). These subpopulations were associated with increased HCC invasion and chemoresistance (115). The core embryonic transcription factor's circuitry (SOX2, c-Myc, Oct4, Nanog) is essential for LCSCs self-renewal (105–107).

Mitogen-activated protein kinase 1 (MAPK1/MEK1) is an oncogene implicated in cancer development and therapy resistance. Active MEK1 signaling is vital for the proliferation and oncogenic potential of LCSC. The interplay between MEK1 and SIRT1 was crucial for upholding the self-renewal and growth of LCSCs. Decreased MEK1 expression in LCSCs reduced the expression of Oct4, c-Myc, SOX2, and Nanog and significantly decreased LCSC self-renewal and proliferation (46). Mechanistically, MEK1 signaling activation increased SIRT1 expression and protein stability and inhibited the proteasomal degradation of SIRT1; this promoted self-renewal and oncogenicity in LCSCs, resulting in poor prognosis of HCC patients (46).

In a cohort of 148 HCC patients, the expression of the MEK1–SIRT1 pathway was strongly correlated with tumor size, vascular and capsular invasion, clinical tumor stage, and poor prognosis (46). MEK1 knockdown in LCSCs isolated from HCC samples lowered SIRT1's half-life, suppressed oncogenicity and self-renewal, and lessened the expression of stem cell markers. However, these results need to be further replicated and validated *in vivo*. Inhibiting SIRT1/MEK1 signaling impedes HCC oncogenesis and should be further explored as a possible therapeutic target (see Figure 1).

### Notch3–SIRT1–LSD1–SOX2 Signaling Pathway

**Figure 2:**
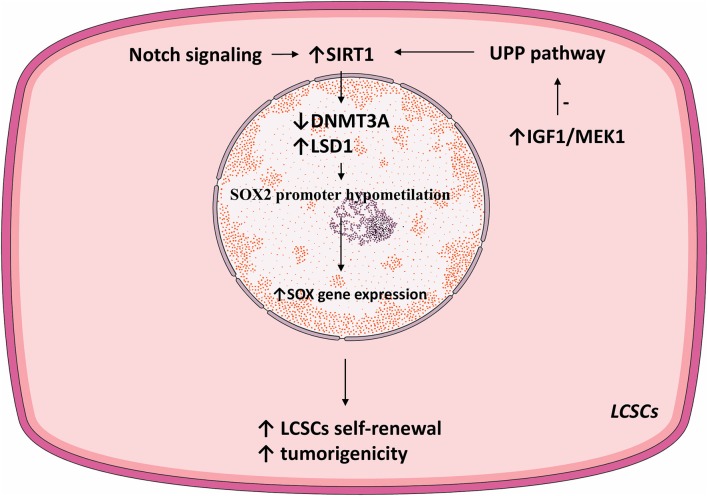
SIRT1-related pathways involved in LCSC proliferation.

Lysine demethylase 1 (LSD1) is an epigenetic regulator responsible for demethylating various histones and controls the pluripotency of stem cells (116–118). LSD1 is overexpressed in HCC cells compared to normal hepatic parenchyma. Moreover, LSD1 is overtly expressed in LCSCs where it directly regulated the transcription of the SOX2 gen, promoted self-renewal and tumorigenesis, and was associated with a poor patient prognosis (119). Similar to the effect of SIRT1 *via* DNMT3A, LSD1 demethylated the SOX2 promoter and consequently increased its expression and improved LCSC stemness. Acetylation inhibits the enzymatic activity of LSD1 and stimulates its degradation *via* UPP. SIRT1 deacetylated LSD1 and thus increased its stability (see Figure 2).

Notch signaling is vital for cell proliferation and survival (120). In HCC, Notch receptors are mostly overexpressed and their ligand expression was associated with aggressive tumor phenotypes (121). Notch promoted HCC development and metastasis through activating the Wnt/β-catenin pathway (122, 123). Moreover, Notch signaling was shown to promote CSC self-renewal. Notch3 signaling induced SIRT1 expression and facilitated LDS1 deacetylation and activated LSD1, consequently promoting LCSC self-renewal. The Notch3-dependent pathway was crucial for LCSC self-renewal and *in vivo* tumor dissemination (see Table 1).

**Table 1:** Molecules involved in the biology of LCSCs and their interaction with SIRT1.

	**Role in HCC**	**Interaction with SIRT1**	**Comments**	**References**
MRPS5	↑LCSCs mitochondrial function and NAD levels	SIRT1 deacetylates MRPS5 and promotes its nuclear localization	Metformin ↓MRPS5	(101)
SOX2	↑LCSCs self-renewal ↑Tumorigenicity	SIRT1 promotes SOX2 expression	SIRT1 inhibitors TV6 and EX-527 ↓SOX2	(88)
MEK1	↑Proliferation and oncogenesis of LCSC	MEK1 ↑ SIRT1 expression in LCSC	↑MEK-SIRT1 expression correlated with HCC metastasis	(46)
LSD1	↑LCSCs self-renewal and tumorigenesis	SIRT1 deacetylates LSD1 and prevents its degradation	↑SOX2 expression	(119)
CPEB1	↓Chemoresistance ↓Stemness ↑Apoptosis	↓SIRT1 expression	CPEB1 expression is ↓ in LCSCs and HCC	(124)

### SIRT1–CPEB1 Signaling Pathway

Cytoplasmic polyadenylation element-binding protein 1 (CPEB1) mediates mRNA translation and negatively regulates HCC stemness and chemoresistance. Moreover, CPEB1 expression was low in HCC and LCSCs when compared to normal hepatocytes (124).

In HCC, CPEB1 upregulation decreased chemoresistance, accelerated doxorubicin-induced apoptosis, inhibited cell migration and self-renewal and decreased tumoral growth while CPEB1 knockdown had the opposite effect.

The 3′ untranslated region (3′UTR) of SIRT1 mRNA presents two cytoplasmic polyadenylation element (CPE) sequences. CPEB1 binds to SIRT1 mRNA and could curtail the poly(A) extremity of SIRT1 mRNA, thus decreasing SIRT1 protein levels. Thus, CPEB1 regulated SIRT1 expression at the post-transcriptional level. Hence, decreased CPEB1 expression may account for SIRT1 overexpression, which in turn promotes LCSCs self-renewal, chemoresistance, and HCC cell spheroid formation (124).

## The Interplay Between miRNAs and SIRT1 in the Development and Treatment of HCC

MicroRNAs (miRNAs/miRs) are endogenous, single-stranded, non-coding regulatory RNAs (131) that exert their biological functions by integrating into the RNA-inducing silencing complex of their target mRNA where they attach to the 3′UTR and either inhibit mRNA translation or induce its degradation (47, 132). The function of multiple miRs was shown to be dysregulated in HCC and in a plethora of other cancers. Depending on the cellular environment and target genes, miRs can function as either oncogenes or tumor suppressors (47, 133, 134). miRs were demonstrated to play a role in HCC development, proliferation metastasis, and therapeutic resistance (121, 135, 136).

### p53-miR-34a–SIRT1 Signaling Pathway

#### 0404

0404, is a DNA-damaging compound with no cytotoxic effects on non-cancerous human hepatocytes. 0404 induced apoptosis and decreased growth in an *in vivo* HepG2 HCC model. However, P53 WT HepG2 cells were more responsive to 0404 compared to the p53 mutant Huh7 cell lines (137).

P53 modulates the transcription of multiple miRs. In turn, numerous miRs target the 3′UTR region of p53 mRNA. Hence, p53 and miRs may form a feedback loop (138).

The miR-34 family is typically silenced in multiple tumors and was identified as the most frequent p53-induced miRs (139, 140). miR-34a was shown to increase p53 transcription and acetylation and induced apoptosis in HCC cells. In HepG2 but not in Huh7 cell lines, 0404 upregulated p53 and miR-34a expression, increased acetylated p53, and downregulated SIRT1 protein expression, which consequently inhibited HCC growth (137). The anticancer mechanisms induced by 0404 and its toxicity and efficacy should be examined *in vivo* on multiple HCC cell lines.

#### Quercetin

Quercetin is a flavonoid with anti-cancer proprieties and low toxicity to non-cancerous cells. Quercetin activated apoptosis and cell cycle arrest in HepG2 and Huh7 cells. However, p53 status determined the sensitivity of HCC cells to quercetin. Namely, in p53 WT cell lines, proliferation was reduced by a significantly lower quercetin quantity, when compared to p53 mutants. HepG2 cells treated with quercetin showcased increased p53 expression, miR-34a activity, and SIRT1 inhibition (140, 141).

SIRT1 deacetylates p53, resulting in the cessation of its activity. miR-34a silences SIRT1 mRNA by binding to its 3′UTR region (142). Quercetin activated p53, which induced miR-34a and consequently silenced SIRT1 mRNA expression, leading to increased p53 acetylation, activity, and consequently apoptosis, thus forming a positive feedback loop. In summary, quercetin activates the p53-miR-34-SIRT1 axis and induces a positive feedback loop that suppresses tumor formation (143).

### miR-34a-IL-24 Oncolytic Adenoviruses

miR-34a expression was shown to be downregulated in multiple cancers including HCC, where it exerts a tumor-suppressive role. In HCC, miR-34 levels were inversely correlated with vascular invasion, necrosis, and histological staging, and low miR-34a expression was associated with decreased overall survival (144).

miR-34a delivery *via* oncolytic adenovirus killed HCC cells *in vitro*, with low toxicity to normal hepatocytes. Importantly, miR-34a expressed in HCC *via* oncolytic adenoviruses, downregulated SIRT1 and Bcl-2 (144) expression, and induced cancerous cytotoxicity (144).

The cytokine IL-24 is known to inhibit tumoral angiogenesis and activates tumoral apoptosis. It was hypothesized that increasing the expression of both IL-24 and miR-34a would provide synergistic therapeutic benefits. Oncolytic adenovirus-mediated transfer of both miR-34a and IL-24 led to a more potent inhibition of HCC cell growth than administering either miR-34a or IL-24 separately. However, the antitumoral mechanisms of the oncolytic adenoviruses used in the abovementioned study are insufficiently understood and its effects on metastasis should also be explored.

### Butyrate–miR-22–SIRT1 Signaling Pathway

Butyrate, a short-chain fatty acid, is produced by the intestinal microbiome *via* anaerobic fermentation and is subsequently absorbed by the hepatocytes (145). Butyrate was shown to induce apoptosis and decrease tumorigenesis in multiple malignancies (146, 147). Butyrate was reported to inhibit SIRT1 gene expression in some types of cancer, although this has not yet been demonstrated in HCC (148).

miR-22 was shown to be downregulated in HCC and its low levels contributed to tumorigenesis (149). miR-22 expression activated apoptosis and inhibited the *in vitro* proliferation of the Huh7 cells. Oppositely, SIRT1 expression was high in Huh7 cells and increased the expression of antioxidants such as superoxide dismutase (SOD), consequently maintaining cell proliferation (40).

In Huh7 cells, butyrate induced miR-22, which directly binds the 3′UTR region of SIRT1 and downregulates its expression; this reduced SOD activity and augmented ROS production, increasing caspase 3 and cytochrome c activity, thus promoting apoptosis (150). Furthermore, by downregulating SIRT1, miR-22 increased PTEN and gsk-3 expression and downregulated β-catenin and p-akt expression and thus may promote apoptosis and decrease HCC proliferation (150).

**Figure 3:**
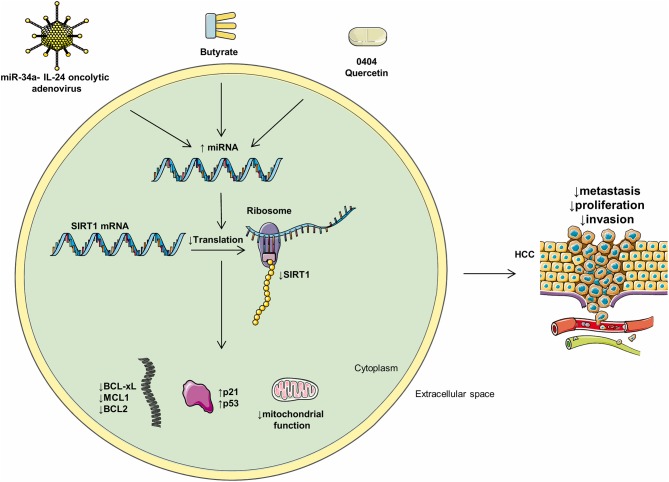
Mediators of miRs and their intracellular effects. Multiple compounds may influence the levels of intracellular miRs and negatively regulate HCC progression. Oncolytic adenoviruses, butyrate, 0404, and quercetin positively regulate multiple HCC miRs and, as a consequence, decrease metastasis, proliferation, and invasion.

However, multiple aforementioned experiments were only performed with 2D cultures or *in vitro* (150, 151). 3D cultures better mimic the *in vivo* environment (152) and it was reported that therapeutic approaches are less effective in 2D cultures when compared to 3D ones. Drug resistance was also reported to be higher in 3D cultures (153–155). Thus, replicating therapeutic interventions performed with monolayer cultures with spheroids, or *in vivo*, may offer a better understanding of their translational potential (see Figure 3).

### miR-133b-Sirt1-GPC3-Wnt/β-Catenin Signaling Pathway

The Wnt/β-catenin pathway is essential for the physiological functioning of the liver (156) and is also implicated in oncogenesis (157). In non-cancerous cells, SIRT1 deacetylates β-catenin, thus constricting it to the cytoplasm and limiting its ability to trigger transcription and induce cell proliferation (25).

The β-catenin gene is commonly mutated in HCC (158). SIRT1 is negatively associated with β-catenin mutation in HCCs, demonstrating that SIRT1 may promote oncogenesis in cancers independent of Wnt/β-catenin.

miR-133b mainly acts as a tumor suppressor and is markedly reduced in a multitude of cancers (159). miR-133b expression was decreased in a majority of HCC biopsies when compared to paired adjacent normal tissue (160). Moreover, miR-133b upregulation in HepG2 cells strongly repressed HCC cell proliferation and invasion and promoted apoptosis (47). Additionally, miR-133b upregulation decreased tumor growth, in nude mice with orthotopic HepG2 cell tumors. miR-133b directly targets and is inversely correlated with SIRT1 in human HCC cells. Enhanced miR-133b expression strongly decreased SIRT1 expression at both mRNA and protein levels. Overall, the anti-cancer effect of miR-133b in HCC cells appears to be achieved through inhibiting SIRT1 expression.

GPC3 stimulates HCC growth *via* Wnt signaling. It has been reported that SIRT1 inhibition decreases the expression of cancer markers AFP and GPC3. Decreased GPC3 and AFP expression indicate the development of an increasingly differentiated cell phenotype and may be beneficial (42). GPC3 is a membrane protein that is extremely overexpressed in HCC and is involved in hepatocarcinogenesis (161–163). GPC3 suppression in HCC cells inhibited proliferation and the expression of anti-apoptotic proteins (Mcl-1, Bcl-2, and Bcl-xL) and also increased TGF-β expression (47). GPC3 also promoted HCC cell EMT (164).

Malignant cells are characterized by inadequate intercellular adhesion and increased cellular motility. E-cadherin is a molecule vital for cell–cell adhesion in epithelial tissues (165). Dysregulation of E-cadherin-modulated intercellular adhesion is observed in human carcinomas and correlates with acquisition of metastatic potential (166). Downregulating SIRT1 decreased GPC3 mRNA and increased the mRNA expression of E-cadherin (42). miR-133b upregulation or GPC3 downregulation repressed GPC3, Mcl-1 Bcl-2, Bcl-xL, and SIRT1 expression and increased the expression of E-cadherin. Moreover, GPC3 downregulation canceled the SIRT1 overexpression-induced inhibition of apoptosis and accelerated invasion and proliferation (47). Additionally, in HCC cells, miR-133b overexpression inhibited GPC3 expression and cell proliferation.

GPC3 interacts with Wnt ligands, consequently stimulating cell migration and proliferation in HCC (167, 168). Activation of the Wnt signaling pathway induces cytoplasmic buildup and nuclear translocation of the transcription factor β-catenin. Intranuclear β-catenin induces the expression of genes that regulate cell differentiation, proliferation, migration, and apoptosis (168–170). SIRT1 upregulation increased the expression of GPC3, which consequently stimulated the Wnt/β-catenin pathway and induced cytosolic accumulation and nuclear translocation of β-catenin. Concluding, miR-133b suppresses cell proliferation and migration and activates cell apoptosis, by inhibiting the Sirt1-GPC3-Wnt/β-catenin signaling pathway (47).

However, SIRT1 was reported to suppress Wnt/β-catenin in multiple malignancies including mouse and human HCCs (171–175). Mechanistically, SIRT1 expression was shown to stimulate β-catenin phosphorylation, which promoted its degradation. SIRT1 regulation of β-catenin is contingent on protein kinase A (PKA). SIRT1 may stimulate PKAs auto-phosphorylation but may also influence PKA through transcriptional regulation of PGC1α–PKA's upstream regulator.

SIRT1 can activate the transcription of PGC1α in HCC cells. PGC1α was demonstrated to elevate cAMP, which, in turn, stimulates the activity of PKA. Thus, SIRT1 may collaterally promote the phosphorylation of PKA and β-catenin through a PGC1α-cAMP-dependent manner (175). SIRT1 also increases βTrCP gene expression. βTrCP is crucial for Sirt1-induced Wnt/β-catenin signaling inhibition. Namely, phosphorylated β-catenin is ubiquitinated by βTrCP and consequently degraded.

Overall, suppressing SIRT1 expression in HCC for therapeutic purposes may activate Wnt/β-catenin signaling and promote tumorigenesis. To short-circuit this potential side effect, the Wnt/β-catenin pathway should be simultaneously inhibited (175).

### miR-449–SIRT1–SREBP-1c Signaling Pathway

Sterol regulatory element binding protein (SREBP)-1c is a transcription factor predominantly localized in adipocytes and hepatocytes, where it regulates lipid synthesis-related gene expression. Abnormal lipid metabolism has been connected to HCC development (65, 176) and SREBP-1c dysfunction is involved in oncogenesis (177–179). SIRT1 regulates lipid metabolism and its activation decreased the expression of hepatic SREBP-1c (180).

miR-449 is part of a miR family that regulates apoptosis and proliferation and may promote tumor suppression *via* downregulating histone deacetylases (181, 182). In Huh7 and HepG2 cell lines, miR-449 directly targets and inhibits SIRT1 mRNA expression, which consequently inhibits SREBP-1c and thus constrains cholesterol and fatty acid biosynthesis (151). Additionally, the SIRT1-SREBP-1c downstream metabolic oncogenes 3-hydroxy-3-methylglutaryl CoA reductase (HMGCR) and fatty acid synthase (FASN) (177, 183) are also downregulated. Overall, miR-449 expression decreases mice HCC xenograft development (151, 184).

Thus, miR-449 inhibited the SIRT1–SREBP pathway which decreased proliferation and DNA synthesis, reduced lipid anabolism, and suppressed tumorigenesis in Huh7 and HepG2 cell lines (151).

### MALAT1–miR-204-5p–SIRT1 Signaling Pathway

The lncRNA metastasis associated lung adenocarcinoma transcript 1 (MALAT1) is decidedly expressed in HCC where it stimulates growth and invasion. MALAT1 activates mechanistic target of rapamycin (mTOR) signaling (185) and enhances the development of HCC CSC (186).

Oppositely to MALAT1, miR-204 promotes apoptosis by activating p53 and inhibiting anti-apoptotic protein Bcl-2 (187). miR-204 also inhibited cancer stemness and EMT and increases chemosensitivity (187, 188). However, MALAT1 expression was negatively correlated with miR-204 levels. MALAT1 directly attaches to miR-204 and negatively regulates its expression (189). SIRT1 appears be a vital intermediary in the interplay between MALAT1 and miR-204. It is known that SIRT1 is vital for HCC EMT, migration, and invasion. SIRT1 is directly targeted and silenced by miR-204 (189). However, SIRT1 and MALAT1 attach to the same miR-204 site; thus, MALAT1 might be in competition with SIRT1 for binding miR-204; this decreases miR-204-induced SIRT1 inhibition. Overall, MALAT1 negatively regulated miR-204 activity and consequently increased SIRT1, which, in turn, induced HCC migration and invasion (189). Inhibiting MALAT1 decreased the aggressive behavior of HCC, which makes it a potential therapeutic target (189).

Moreover, it was shown that miR-204-5p expression was decreased in multiple human HCC cell lines (189). Decreased miR-204-5p levels were associated with HCC metastasis and poor patient outcome. In HCC cell lines, miR-204-5p binds to the 3′UTR region of SIRT1 and consequently decreases its expression at both mRNA and protein levels. Likewise, in these same cell lines, miR-204-5p was inversely associated with SIRT1 expression. miR-204-5p decreased the invasion and survival of HCC cells by reducing SIRT1 expression and protein levels both *in vivo* and *in vitro* (190). Overall, miR-204-5p induced post-transcriptional inhibition of SIRT1 in multiple human HCC cell lines. Importantly, miR-204-5p overexpression strongly downregulated both SIRT1 mRNA and protein levels in HCC cells and thus attenuated tumoral growth. As such, miR-204-5p is a potential diagnostic marker and increasing its expression and activity in HCC cells is a therapeutic option that needs to be further explored (190).

### miR-486

miR-486 downregulation may be a hallmark of HCC development and contributed to the differentiation of LCSCs into HCC cells (43). miR486 was shown to be strongly downregulated in HCC samples and in LCSCs (43).

On the contrary, SIRT1 expression was increased in LCSCs and maintained the tumorigenic and self-renewal proprieties of LCSCs *in vivo*, and was inversely correlated with miR-486 levels in LCSCs (43). miR-486 directly targets and strongly suppresses SIRT1 expression and decreased the tumorigenic and chemo-resistant properties of LCSCs and suppressed HCC invasion and tumorigenicity (43).

Overall, this further validates the role of SIRT1 as a promoter of HCC development, invasion, and recurrence, in part through maintaining the stemness of CSCs (43).

### miR-29c

Decreased levels of the miR-29 family were associated with poor HCC survival. The miR-29 family have tumor-suppressive roles by targeting Mcl-1 and Bcl-2. Relevantly, miR-29c exerts tumor-suppressing functions by inhibiting hepatocytic SIRT1. Ectopic miR-29c expression decreased SIRT1 expression and consequently repressed cell proliferation. miR-29c directly targets and suppresses SIRT1 mRNA translation in hepatocytes (191).

miR-29c was shown to be strongly downregulated in HCC biopsies and correlated with poor patient prognosis. The 5-year survival rate of HCC patients with low miR-29c expression was pointedly inferior to that of HCC patients with high miR-29c expression. Future interventions that upregulate miR-29c in HCC models may provide insights into a more efficient management of HCC (191).

### miR-29a

Decreased miR-29a expression is common in HCC, where it promoted metastasis and, as a predictor of early post-surgical recurrence, diminished overall survival and disease-free survival (192). miR-29a suppressed HCC proliferation. Tellingly, miR-29a was strongly downregulated in HCC tissue biopsies when compared with adjacent normal tissue. miR-29a upregulation suppressed HCC cell proliferation and colony formation. Mechanistically, miR-29a increased p21 expression and decreased CDK4 and CyclinD1 expression, consequently suppressing cell cycle progression; it also targeted the 3′UTR region of SIRT1 mRNA and decreased SIRT1 mRNA and protein expression consequently overturning HCC cell proliferation (192). Oppositely, SIRT1 overexpression decreased the protective effects exerted by miR-29a. Overall, data suggests that miR-29a may serve both as a prognostic marker and as a therapeutic target for HCC suppression.

### miR-138

It has been confirmed that miRs have suppressive or promotive effects on tumor metastases and invasion and impact HCC progression (193).

miR-138 functions as a tumor suppressor and it was found to be downregulated in multiple cancers (194). In HCC cells, miR-138 suppresses cell invasion and proliferation. Interestingly, miR-138 expression levels were inversely correlated with SIRT1 mRNA levels in HCC tissues (193). Upregulation of miR-138 expression downregulated SIRT1 at the level of mRNA and protein levels. miR-138 binds to the 3′UTR unique complementary site of the SIRT1 gene and directly inhibits SIRT1 expression, which leads to hindered HCC proliferation, migration, and invasion (193). miR-138 was inversely correlated with SIRT1 mRNA in HCC tissues.

Multiple studies showed that miR-138 expression was downregulated in a majority of examined HCC samples compared with peritumoral non-cancerous tissue (193). SIRT1 is overexpressed while miR-138 levels are decreased in HepG2, SMMC7721, Bel7404, and HCCM3 compared to the normal hepatic cell line L02 (193).

Tellingly, increasing miR-138 expression in HepG2 and SMMC7721 cell lines inhibited their proliferation (193). This suggests that decreased miR-138 expression was associated with increased SIRT1 mRNA expression in HCC (193). This validates the role of miR-138 in HCC proliferation and metastasis (195).

### miR-34a

miR-34a is a tumor suppressor in breast, colon (196), and a plethora of cancers. miR-34a expression was correlated with HCC metastasis (197). miR-34a downregulated c-Met expression and consequently inhibited HCC invasion and migration (198), which have oncogenic or tumor suppressor functions (199).

miR34a expression was decreased in Hep3B and Huh7 cells when compared to normal hepatocyte cell lines. Inducing miR-34a overexpression in Hep3B and Huh7 significantly decreased cellular invasion and migration (200). Moreover, miR-34a overexpression also decreased SIRT1 mRNA and protein levels and increased acetylated p53. Thus, miR-34a overexpression downregulated SIRT1 expression, which increased acetylated p53 levels and consequently suppressed HCC metastasis (200).

All in all, data suggest that the post-transcriptional overexpression of SIRT1 may be promoted by loss of suppressive mRNAs that normally target and inhibit its expression.

## The Role of SIRT1 in the Metastasis of HCC

Metastases are a great contributor to HCC morbidity (12). SIRT1 overexpression in HCC samples correlated with advanced tumor stage and increased incidence of portal vein tumor thrombus. Moreover, SIRT1 overexpression in HCC facilitates invasion and proliferation and suppresses apoptosis (15, 126, 191). SIRT1 also increased the invasiveness and motility of human HCC cells and was necessary for HCC metastasis *in vitro*. Importantly, silencing SIRT1 reduced the aforementioned metastatic characteristics of human HCC cells (54). Accelerated cancer invasiveness is associated with increased mitochondrial activity, oxygen consumption, and ATP production (201).

One way in which SIRT1 promotes HCC metastasis is by its interaction with peroxisome proliferator–activated receptor γ coactivator 1α (PGC-1α). PGC-1α is a transcriptional co-activator that promotes mitochondrial biogenesis and respiration (19, 202). PGC-1α expression was demonstrated to enhance the invasion and migration of HCC cells (54). In HCC samples, SIRT1 overexpression was highly correlated with PGC-1α upregulation. Ectopic SIRT1 expression upregulated PGC-1α in HCC cells. Moreover, SIRT1 physically interacts with, deacetylates, and activates PGC-1α. SIRT1-induced PGC-1α increased mitochondrial copy numbers and mass, cellular ATP levels, DNA transcript levels, and mitochondrial biogenesis, which boosted the migration and invasion of HCC, thus promoting cancer dissemination (54, 203).

PGC-1α-induced mitochondrial biogenesis and oxidative phosphorylation were crucial for HCC metastasis (204)SIRT1 knockdown in HCC cells reduced the expression of mitochondria biogenesis-related genes, diminished mitochondrial mass and copy number, intracellular ATP, and mitochondrial DNA transcript levels. These changes led to decreased intracellular ATP production and impaired HCC metastasis (54).

The epithelial-to-mesenchymal transition (EMT) was shown to promote HCC metastasis (205). Through EMT, epithelial cells gain mesenchymal-like characteristics such as reduced intercellular junctions, enhanced invasiveness and motility, chemoresistance, and decreased polarization (206). SIRT1 expression promoted migration and invasion of HCC cell lines and metastasis in an *in vivo* xenograft mice model by activating EMT (45).

However, other reports suggest that SIRT1 expression was not occasionally activated in human HCC cell lines, suggesting that SIRT1-arbitrated metastasis did not implicate EMT (54). The difference may be attributed to the different HCC models used in the experiments.

Combined, these results suggest that SIRT1 expression promotes progression, metastasis, and invasion of HCC. SIRT1 knockdown decreased migration and invasion of HCC cells *in vitro*, decreased HCC invasion and metastasis *in vivo* (45, 54), and impaired mitochondrial function and biogenesis, suggesting that the SIRT1/PGC-1α axis may be a viable therapeutic target for decreasing metastasis (54).

## Implications of SIRT1 in the Treatment and Chemoresistance of HCC

HCC is a chemo-refractory cancer (207). Multidrug resistance (MDR) is partly incriminated for HCC metastasis and recurrence. The ATP binding cassette (ABC) transporters are involved in the cellular efflux of chemotherapeutics. ABC overexpression is partly incriminated for HCC MDR (208). The ABC transporters, P-glycoprotein, or multidrug resistance protein 1 (P-gp, MDR1) and multidrug resistance protein 3 (MRP3) are important for HCC chemotherapy (209–211).

SIRT1 expression was shown to stimulate oncogenesis and promote MDR in HCC (40, 212). SIRT1 overexpression upregulates MDR1 in HepG2 cells (213). Contrarywise, silencing FOXO1 or SIRT1 accentuates the cellular uptake of chemotherapeutics and reinstates HCC chemosensitivity. For instance, SIRT1 knockdown in human HCC cells enhanced doxorubicin-induced chemosensitivity and apoptosis (15).

Acetylated p53 promotes tumor suppression by interacting with its downstream targets. SIRT1 deacetylates both p53 and FOXO1, consequently suppressing their ability to induce apoptosis and cell growth arrest (214, 215).

FOXO1's activity is controlled by post-translational interventions, including acetylation, ubiquitination, and phosphorylation. SIRT1 deacetylates FOXO1 and thus promotes its nuclear localization and increases the FOXO-dependent transcription of stress-response genes (216, 217). Specifically, FOXO1 binds to the MDR1 gene promoter and increases MDR1 gene transcription.

Deleted in Liver Cancer-1 (DLC-1) is an established tumor suppressor that has important roles in cell motility and signal transduction pathways. Akt regulates DLC-1 activity by post-translational modifications. SIRT1, by inhibiting the PI3K/Akt pathway, could increase DLC-1 expression and thus promote HCC cell motility (218).

### HULC/USP22/SIRT1/Autophagy Pathway

Autophagy is involved in the chemoresistance of cancer cells (219). Cisplatin, sorafenib, and 5-FU can induce autophagy in HCC cells, thus decreasing apoptosis and chemosensitivity (220). Long noncoding RNAs (lncRNAs) are transcripts involved in regulating gene expression. lncRNA HULC (highly upregulated in liver cancer) is involved in HCC chemoresistance and autophagy. In HCC biopsies and cell lines, SIRT1 and HULC are both aberrantly upregulated.

Oxaliplatin and 5-FU upregulated HULC expression in HCC cells. In turn, HULC strongly increased USP22 protein levels and promoted SIRT1 deubiquitylation, consequently decreasing the UPP-mediated degradation of SIRT1 and increased its protein stability. miR-6825-5p, miR-6886-3p, and miR-6845-5p bind to the 3′UTR region of USP22 mRNA and thus decrease its levels. HULC downregulates the abovementioned miRs and strongly upregulates USP22. HULC-induced SIRT1 upregulation enhanced the deacetylation of key autophagy components such as Atg7 and Atg5 and triggered autophagy, which decreased HCC chemosensitivity. In summary, the HULC/USP22/SIRT1 pathway induces protective autophagy and decreases HCC chemosensitivity. *In vivo* knockdown of HULC or SIRT1 sensitizes HCC to oxaliplatin. Thus, combining chemotherapy with HULC or SIRT1 inhibitors in MDR HCC is a therapeutic option that needs further exploration (see Table 2).

**Table 2:** Therapeutic substances that interfere with SIRT1-related pathways in HCC.

**Medication**	**Targeted signaling pathways and biological processes**	**Effects on HCC**	**Type of study**	**References**
Oxaliplatin and 5-FU	HULC-USP22-SIRT1	↑Autophagy ↑HCC chemosensitivity	*in vivo*	(125)
EX-527 and cambinol	SIRT1 inhibitors	↑Apoptosis ↓Cell migration ↓ Tumoral growth	*in vivo*	(126)
Metformin	↑AMPK activity ↑Acetylated p53 and p21	↑Senescence ↑Apoptosis ↓Proliferation	*in vitro* and *in vivo*	(127)
Gallotannin	↑AMPK phosphorylation ↓SIRT1 expression	↓Colony formation ↑Cytotoxicity ↑Senescence	*in vitro* and *in vivo*	(128)
Ku0063794 and Everolimus	↓SIRT1 expression	↓Proliferation ↓Autophagy ↑Apoptosis	*in vivo, ex vivo*, and *in vitro*	(129)
2-Unsubstituted 4,11-diaminoanthra[2,3-b]furan-5,10-dione derivatives	↓tNOX ↓Intracellular NAD+ ↓SIRT1 ↓Acetylated p53	↑ Apoptosis ↓Cell migration	*in vitro*	(130)

### Cambinol and EX-527

EX-527 and cambinol are cytotoxic to and trigger apoptosis in both 2D and 3D HCC cultures (42). Cambinol and EX-527 are SIRT1 inhibitors with antitumor effects that decrease MDR1 mRNA expression in a p53 status- and dose-dependent manner. This suggests that their administration and compatibility with other chemotherapeutics should be adjusted according to the particular genotype of the HCC treated (221).

Cambinol or EX-527 decreased SIRT1 activity and protein levels and thus increased apoptosis, reduced cell migration, and decreased the growth and viability of HCC cell spheroids (60, 126). EX-527 increased the acetyl-p53/p53 ratio in HCC cells and promoted apoptosis. Surprisingly, cambinol decreased the acetyl-p53/p53 radio in Huh7 cells, and increased p53 protein levels. The reason for this discrepancy and the consequences of cambinol on p53 acetylation should be further explored in multiple HCC models. However, both Ex-527 and cambinol decreased FOXO1 expression and increased FOXO1 acetylation, which consequently decreases P-gp in 2D HCC cultures. Whether this reduces chemoresistance should be further explored in HCC spheroids or *in vivo* models.

SIRT1 downregulation suppresses MRP1 and increases intracellular concentration of Adriamycin in MDRHCC. Downregulating SIRT1 *via* shRNA decreases MRP3 and MRP1 protein levels. However, the effects of cambinol and Ex-527 on those proteins in HCC have not yet been explored.

### Sorafenib

Sorafenib is used in the treatment of advanced HCC. Notably, HCC cells resistant to sorafenib showcased increased MRP3 and P-gp expression. Thus, combining cambinol or EX-527, which downregulates P-gp and MRP3 in HepG2, with conventional chemotherapeutics may offer new treatment options against MDRHCC. Surprisingly, cambinol and EX-527 induced MRP3 and P-gp expression in Huh7 cells. Since HepG2 are p53 WT while Huh7 are p53 mutant, it was suggested that this may at least partially account for the discrepancy.

Therapy response may depend on p53 status, which appears to influence the expression of ABC transporters in MDRHCC. Therefore, the p53 status of each HCC has to be considered when evaluating for susceptibility to chemotherapy. This undermines the importance of characterizing the chemosensitivity of multiple HCC types and personalizing treatment accordingly.

### USP22/SIRT1/AKT/MRP1 Signaling Pathway

Ubiquitin-specific protease 22 (USP22) is part of a subfamily of deubiquitinating enzymes and a CSC marker. USP22 is exceedingly expressed in some multidrug resistant human HCC cell lines (MDRHCC) where it decreases sensitivity to 5-fluorouracil (5-FU), doxorubicin, and methotrexate; specifically, it reduced the intracellular concentration of doxorubicin by promoting efflux pump activity and inhibited 5-FU-induced apoptosis. Similarly, SIRT1 expression in MDRHCC decreased intracellular doxorubicin concentrations and promotes resistance to 5-FU.

Inhibiting USP22 in MDRHCC strongly decreased ABCC1 expression [encodes MRP1 (resistance-associated protein 1)] and thus increased intracellular doxorubicin concentrations, but only dimly influenced ABCB1 expression (encodes P-gp). Thus, UPP22 may predominantly induce MDR *via* MRP1.

USP22 deubiquitinated SIRT1 (222, 223) and thus increased SIRT1 protein levels, which deacetylated and thus activated the PI3K/AKT pathway (224) and consequently increased MRP1s expression (225), which promoted MDR in HCC. However, this mechanistic chain needs to be further validated by other experiments. In 168 HCC biopsies, the protein expressions of MRP1 and USP22 were strongly correlated. Inhibiting the PI3K/AKT pathway in MDRHCC suppressed MRP1's expression and promoted 5-FU-induced apoptosis (226). SIRT1 inhibition increased the sensitivity of MDRHCC to 5-FU and increased intracellular concentration of doxorubicin. Simply put, USP22 may activate the SIRT1–AKT–MRP1 pathway and consequently promote MDR in human HCC cells (226). Future studies should explore the relationship between USP22 and other proteins involved in MDRHCC such as MRP1 and cytochrome P450, which is involved in the hepatic metabolism of xenobiotics.

### SIRT1–YAP Signaling Pathway

SIRT1 stimulated the transcription of MKK3 and Yes-associated protein (YAP), which in turn promoted the nuclear localization of p38. SIRT1 activated p38 and consequently promoted HCC development (44). In HCC cells, SIRT1 expression activated YAP2 transcriptional activity and also accentuated the interaction between YAP2 and the transcriptional cofactor TEAD4, and thus promoted the transcription of their downstream targets, which consequently accelerated HCC cell growth and survival. SIRT1 deacetylates YAP2 both *in vitro* and *in vivo*, which upregulated the YAP2/TEAD4 axis and promoted HCC cell proliferation (60).

Treatment with cisplatin accentuated the interaction between SIRT1 and YAP2 and promoted the expression of the YAP2 downstream genes. As a response to cisplatin, YAP2 translocates to the nucleus where it is deacetylated by SIRT1, which consequently protects HCC cells from cisplatin-induced apoptosis. Overall, both YAP2 and SIRT1 confer protection against cisplatin (60). Silencing SIRT1 inhibited the nuclear translocation of YAP2 and promoted sensitivity to cisplatin.

Since SIRT1 inhibition induced cytostatic effects in HCC (42), combining it with cytotoxic chemotherapeutics should be considered. Moreover, SIRT1 inhibiting compounds used for the purpose of treating HCC should selectively target tumoral SIRT1 and spare normal hepatocytes in order to preserve liver function.

### AMPK–SIRT1–p53 Signaling Pathway

By targeting transcription factors such as p53 and FOXO, SIRT1 suppressed cellular differentiation and senescence and may promote HCC growth (227). Mutual regulation ensues among p53 and SIRT1. SIRT1 deacetylates and inactivates p53 while acetylated p53 downregulates the translation of SIRT1 *via* miR-34a (142). Silencing SIRT1 in HCC cell cultures increases acetylated p53 and promotes growth arrest (41). This relationship is further validated by nicotinamide (NAM), a SIRT1 inhibitor. In mice treated with NAM, p53 presented increased acetylation, which led to decreased HCC oncogenesis. Importantly, the protective effects of NAM were attributed to the inhibition of SIRT1, not to NAM's antioxidant effect.

In HCC cells, SIRT1 deacetylates p53, thus repressing cellular senescence and apoptosis, and promotes tumorigenesis (63, 228). SIRT1 knockdown in HCC cells increased acetylated p53, decreased proliferative activity activated senescence, and induced a more differentiated cellular state (26, 42). However, the response to silencing SIRT1 may depend on the p53 status of HCC cells. Silencing SIRT1 in p53 WT HepG2 cells increased AMPK phosphorylation, reduced phospho-mTOR, and promoted G1 phase arrest (41, 63). However, silencing SIRT1 in p53 mutant HCC decreased phospho-AMPK and increased mTOR phosphorylation, which stimulated HCC tumorigenesis. Moreover, some reports indicate that inhibiting SIRT1 prevented cell proliferation irrespective of the p53 status of the HCC cells (42). The dynamic between the p53 status of HCC cells and how it affects SIRT1 inhibition is not yet clear.

5′ AMP-activated protein kinase (AMPK) is an enzyme implicated in glucose and fatty acid uptake; its activation stimulates hepatic fatty acid oxidation, ketogenesis, and lipogenesis (229). AMPK is a downstream target of tumor suppressor LKB1. The LKB1–AMPK pathway is vital for the suppression of mTOR signaling. mTOR signaling is overactive in multiple solid tumors and modulates cell proliferation (230). The AMPK-α2 subunit was shown to be strongly downregulated in HCC compared with the corresponding normal hepatic tissue and was correlated with poor patient prognosis.

In HCC cells, AMPK-α2 and SIRT1 are co-localized in the nucleus where they directly interact. AMPK phosphorylates SIRT1 at Thr344 and thus inhibits its deacetylase activity and substrate binding capacity and consequently maintains acetylated p53 levels, which promote apoptosis. Thus, AMPK promotes p53 acetylation and exerts antioncogenic functions in HCC (231).

### Metformin

Inducing senescence may be a viable strategy for the chronic management of HCC, since it has fewer negative consequences than therapies that activate apoptosis (232, 233).

Metformin is a drug primarily used for the treatment of type 2 diabetes; it also has antitumoral effects in HCC (127, 234). A low dose of metformin strongly suppressed HCC growth *in vivo* and *in vitro* by inducing senescence, inhibiting proliferation, and activating apoptosis (127, 233). Metformin promoted phosphorylation and activated AMPK, which in turn phosphorylated SIRT1 and disabled its enzymatic activity. Consequently, levels of acetylated p53 and p21 were increased ensuing HCC senescence (127). Additionally, AMPK phosphorylation induced by metformin inactivated mTOR in p53 mutant HCC and negatively regulated oncogenesis (63). These results need to be replicated with a larger variety of HCC cell lines (233).

However, both metformin and AMPK modulate NAD+ metabolism and can induce SIRT1 activity (127, 235, 236). Notably, SIRT1 can be phosphorylated at multiple sites that promote different phenotypes. Nevertheless, in HepG2 cells cultured in a high glucose medium, metformin activated both AMPK and SIRT1 and amplified p53 deacetylation contributing to p53 degradation (237). Specifically, metformin primarily targeted AMPK, which activated SIRT1. In this model, both AMPK and SIRT1 were required for metformin-induced p53 degradation (237).

### Gallotannin

Gallotannin is a plant-derived compound with anticancer effects (125). Treating HCC cells with gallotannin *in vitro* resulted in reduced colony formation, amplified cytotoxicity, increased senescence, impaired autophagy, upregulated p21, and promoted cell death. In a mouse xenograft model, gallotannin decreased tumor growth (128). Mechanistically, gallotannin activated AMPK phosphorylation and decreased SIRT1 expression in both *in vitro* and *in vivo* HCC models (128).

### Everolimus and Ku0063794

The PI3K/AKT/mTOR signaling pathway is involved in the development of multiple malignancies, including HCC (129).

Abnormal mTOR signaling is present in up to 48% of HCC patients and is associated with a meager prognosis. Everolimus is an mTOR complex 1 (mTORC1) inhibitor. However, targeting both mTORC1 and 2 is pivotal for evading drug resistance (129). Administering everolimus with Ku0063794, an mTORC2 inhibitor, produced a potent antioncogenic effect in HCC cells (130). mTOR and SIRT1 both regulate autophagy (130). SIRT1 may promote autophagy *via* deacetylating transcription factors such as E2F1, FOXO1, histone H4, and p53, which subsequently induce autophagy-related genes (238). Moreover, inhibiting mTOR can also activate autophagy (130, 239).

In HepG2 cells, combining Ku0063794 with everolimus decreased autophagy and inhibited SIRT1 expression, whereas individual monotherapy with either of the compounds did not inhibit SIRT1 and promoted autophagy (130, 240). Blocking autophagy stimulated apoptosis, decreased proliferation, and inhibited SIRT1 expression. This suggests that autophagy may promote survival in HCC cells (130).

Overall, combined use of Ku0063794 and everolimus downregulated autophagy by decreasing SIRT1 and consequently promoted antioncogenic effects in HepG2 cells (130). This experiment should be further validated with other HCC cell lines and by using spheroids or *in vivo* models. Moreover, testing whether mTORC1/2 inhibitor AZD8055 also inhibits autophagy would further validate the result obtained with Ku0063794 and everolimus in HepG2 cells (130).

In summary, *in vivo, ex vivo*, and *in vitro* results with HCC cells confirmed that combined Ku0063794 and everolimus therapy was superior to administering either compound alone as indicated by their increased reduction of cell invasion, migration, proliferation, and increased EMT inhibition (240). EMT inhibition was partly produced by decreased SIRT1 levels. Thus, combining everolimus with the mTORC1/2 inhibitor Ku0063794 provides potent anticancer effects (240).

### tNOX-SIRT1-p53

First-line HCC treatment compounds such as doxorubicin are associated with systemic toxicity, inefficacy, and chemoresistance (10, 11). Recently, new anti-cancer compounds with high antiproliferative activity against chemoresistant cells have been developed (241, 242). The human tNOX gene encodes for a protein that is expressed in multiple solid malignancies where it is crucial for cellular migration and proliferation. tNOX converts reduced NADH to oxidized NAD. Importantly, tNOX inhibition reduces intracellular NAD concentration, which influences SIRT1 function (241, 243, 244). Suppression of tNOX by a multitude of agents activated apoptosis and diminished malignant cell growth (244, 245).

Two 2-unsubstituted 4,11-diaminoanthra[2,3-b]furan-5,10-dione derivatives promoted apoptosis in human HCC cells in a tNOX-dependent manner. In p53 WT HCC cells, these anti-cancer compounds bound and downregulated tNOX, which decreased intracellular NAD, and consequently suppressed SIRT1 activity. Decreased SIRT1 activity led to increased p53 acetylation and activation, which upregulated its downstream target, pro-apoptotic Bak and thus increased apoptosis (246). Augmented p53 acetylation promoted by SIRT1 inhibition was also associated with activation of PUMA, which upregulates Bak and prompts apoptosis (246, 247).

tNOX reduction reestablished non-cancer phenotypes, such as decreased migration, and amplified sensitivity to stress-induced apoptosis. Accumulating evidence suggests that suppressing tNOX may improve patient prognosis (246, 248–250).

c-Myc was demonstrated to be an HCC initiating oncogene (251). C-Myc expression is associated with HCC progression and poor patient prognosis (26, 252). In HCC cells, SIRT1 induced the expression of oncogenic c-Myc, which in turn increased β-catenin mRNA and protein expression and amplified the transcription and expression of the β-catenin target genes survinin and cyclin D1. Therefore, SIRT1 overexpression promoted oncogenesis *via* c-Myc activation (39, 246) and protected against p53 induced apoptosis (26).

c-Myc was shown to increase SIRT1 through transcriptional and post-transcriptional regulation. The SIRT1-c-Myc axis impacts cellular growth; however, the result of this interaction is still controversial (253–255).

## Concluding Remarks

HCC accounts for immense mortality rates worldwide and poses difficult therapeutic problems. The therapies used today are inefficient at managing late-stage disease or metastasis. A better understanding of the underlying mechanisms that promote HCC development, metastasis, and chemoresistance may enable the development of more efficient therapeutic protocols. SIRT1 mediates LCSCs stemness, HCC metastasis, and chemoresistance. Targeting SIRT1 either to hinder the progression and metastasis of HCC or to decrease LCSCs stemness may be a viable therapeutic option. Directly inhibiting SIRT1 *via* miRs, exogenous compounds, or combining conventional chemotherapeutics with tumor-selective SIRT1 inhibitors may improve treatment outcomes. However, a better understanding of the biology of SIRT1 in HCC is needed in order to efficiently inhibit related pathways and constrain HCC development.

## Author Contributions

IB-N and CC designed the study and approved the final version of the manuscript. MF, A-AG, and DG wrote the article, while CI and AI selected the most relevant articles to be included in the paper. The figures were created by MF and DG and tables were made by A-AG.

### Conflict of Interest Statement

The authors declare that the research was conducted in the absence of any commercial or financial relationships that could be construed as a potential conflict of interest.
